# *In vitro* antimalarial susceptibility and molecular markers of drug resistance in Franceville, Gabon

**DOI:** 10.1186/1471-2334-12-307

**Published:** 2012-11-15

**Authors:** Rafika Zatra, Jean Bernard Lekana-douki, Faustin Lekoulou, Ulrick Bisvigou, Edgard Brice Ngoungou, Fousseyni S Toure Ndouo

**Affiliations:** 1 Unité de Parasitologie Médicale (UPARAM), Centre International de Recherches Médicales de Franceville (CIRMF), B.P., 769, Franceville, Gabon; 2 Département de Parasitologie-Mycologie Médecine Tropicale, Faculté de Médecine, Université des Sciences de la Santé, B.P., 4009, Libreville, Gabon; 3 Département de Santé Publique et de Médecine Légale et du Travail, Faculté de Médecine, Université des Sciences de la Santé, B.P., 4009, Libreville, Gabon

## Abstract

**Background:**

Malaria remains a major public health problem, due largely to emergence and widespread *P. falciparum* drug resistance. WHO recommends artemisinine combination based therapy (ACT) to overcome *P. falciparum* drug resistance, but reports of declining ACT efficacy have been published. A thorough understanding of the molecular bases of *P. falciparum* resistance to existing drugs is therefore needed. The aims of this study were to analyze the *in vitro* sensitivity of *P. falciparum* field isolates from Franceville, Gabon, to chloroquine (CQ), mefloquine (MF), dihydroartemisinine (DHA) and monodesethylamodiaquine (MDAQ), and to investigate polymorphisms associated with drug resistance.

**Methods:**

We conducted a cross-sectional study of 53 field isolates. Field isolates sensitivity to CQ, MF, DHA and MDAQ was assessed using the colorimetric DELI test. The *Pfmdr1* codons 86 and 1246, *Pfcrt* (haplotype codon 72 to 76) and the *PfATPAse6* codons 110 and 2694 were analysed by PCR-RFLP. Associations between drug sensitivity and parasite gene polymorphisms were evaluated with the Chi square test, and routine hematological parameters were analyzed with Fisher’s exact test implemented with Epinfo software. In all statistical tests, significance was assumed at p<0.05.

**Results:**

A total of 46 *P. falciparum* isolates were successfully cultured *in vitro* and their sensitivity was tested. The proportions of isolates resistant to CQ, MF and MDAQ were 43.5%, 23.4% and 56.5%, respectively. Some isolates (23.9%) had DHA IC_50_ values higher than 10 nM. The median IC_50_ values were 71.67 (interquartile range (IQR, 1–438.2), 6.59 (IQR, 0.08-96), 64.79 (IQR, 0.09-448) and 6.45 nM (IQR, 0.09-23) for CQ, MF, MDAQ and DHA, respectively. The strongest correlation between diminished DHA sensitivity and MF resistance was observed (r^2^=0.73), followed by correlation between diminished DHA sensitivity and CQ resistance. Cross-resistance between CQ and MF was also observed. The prevalence of the 86Y and 1246Y mutations in *Pfmdr1*, 76T in *Pfcrt*, and 110A and 2694T in *PfATPase6* was respectively 42% and 17.1%, 97.8%, and 0% and 22.2%.

**Conclusion:**

These high levels of antimalarial drug resistance in Franceville, Gabon, call for reinforced surveillance of drug efficacy.

## Background

Despite increased funding for control programs, malaria remains a major public health problem, with approximately 781 000 deaths in 2009 [1]. The reasons for this persistence include the lack of an efficacious vaccine, vector resistance to insecticides, and parasite resistance to antimalarial drugs. Drug resistance arises rarely but spreads relatively quickly. *P. falciparum* drug resistance is associated with the emergence of specific parasite genotypes [2-4] and single-nucleotide polymorphisms (SNPs) in parasite genes, including the chloroquine transporter gene *Pfcrt*[5,6]. Higher-level CQ resistance results from other SNPs and is negatively associated with the copy number of the *P. falciparum* multidrug resistance 1 gene *Pfmdr1*[7-9]. *Pfmdr1* polymorphisms also confer resistance to other antimalarial drugs, including mefloquine, lumefantrine, and quinine [9-11]. In areas where drug resistance is prevalent, WHO has recommended artemisinin-based combination therapy (ACT) for the treatment of uncomplicated *falciparum* malaria [12], but several reports have shown the selection of certain genotypes following ACT treatment [13-15]. Of particular concern are the results of a *Pfmdr1* allelic replacement study in which various polymorphisms were found to reduce artemisinin susceptibility in cloned parasite lines [9].

Gabon is a hyperendemic country where resistance to CQ, amodiaquine and sulfadoxine-pyrimethamine is widespread [16-19]. In 2003 the National Malaria Control Program adopted the ACT strategy to treat uncomplicated malaria, instead of CQ and other monotherapies, in line with WHO recommendations. The ACTs most widely used in Gabon are artemether-lumefantrine (AL) and artesunate-mefloquine (AM). Subsequently, the prevalence of malaria among febrile children fell from 30% to 13% between 2004 and 2008, and the mean age of children with malaria rose from 24 to 41 months at the country’s biggest hospital, Centre Hospitalier de Libreville (CHL) [17,20]. However, some ACT treatment failures have been observed (Kombila M et al. unpublished data). In Franceville, a town of 60 000 inhabitants situated in south-eastern Gabon, uncomplicated malaria has been treated with AL and AM since 2005. In this region, the few available data show prevalence rates of about 50% for CQ resistance, 21.1% for MF resistance, and 0% for quinine resistance [18], but the prevalence of *Pfcrt* and *Pfmdr1* genotypes is poorly documented. We recently found that the change in national antimalarial policy had led to the selection of N86 *Pfmdr1* strains, whereas the prevalence of other polymorphisms in *Pfmdr1* and *Pfcrt* remained stable [21]. Some data suggest a decline in the *in vitro* efficacy of artemisinin and its derivatives and also that of other drugs used in ACT regimens [22-24].

The aims of this study were 1) to determine the *in vitro* sensitivity of clinical *P. falciparum* isolates to chloroquine, mefloquine, monodesethylamodiaquine and dihydroartemisinin, and 2) to determine the prevalence of polymorphisms in the resistance-associated genes *Pfmdr1*, *Pfcrt* and *PfATPase6* in Franceville, Gabon.

## Methods

### Clinical isolates

After obtaining informed consent from the parents or guardians, clinical isolates were collected from children infected with *P. falciparum* in both Franceville hospitals (Centre Hospitalier Régional Amissa Bongo and Hôpital de l’amitié sino-gabonaise) from October 2009 to May 2010. Giemsa-stained thin and thick blood smears were examined for *P. falciparum* mono-infection. Parasite density was determined according to Lambarené's methods [25]. If parasitemia was higher than 0.5%, the sample was diluted with uninfected group O^+^ erythrocytes before incubation with the drugs. The isolates were studied within 24 h following harvest. The study was approved by the Human Ethics Committee of Centre International de Recherche Médicale de Franceville.

### Drugs

CQ and mefloquine (MF) were obtained from Sigma Aldrich. Dihydroartemesinine (DHA) and monoadhesylamodiaquine (MDAQ) were a gift from B. Pradines (IMTSSA, Marseille, France). The antimalarial drugs were prepared in methanol and serially diluted in complete culture medium (RPMI 1640, Gibco-BRL, Gaithersburg, MD) containing 35 mM HEPES (Sigma, St. Louis, MO), 24 mM NaHCO3, 0.5% Albumax (Gibco-BRL), 1 mg/liter hypoxanthine (Sigma), and 5 μg/ml gentamicin (Gibco-BRL). Cultures were synchronized with 5% sorbitol [26]. The following final drug concentrations were used: 2000, 1000, 500, 250, 125, 62.5, 31.25, 15.625, 7.8, and 3.9 nM CQ; and 1000, 100, 50, 25, 10, 5, 1 and 0.1 nM DHA, MDAQ and MF.

### Maturation of *P. falciparum* isolates

Maturation assays were performed in 96-well tissue culture plates. Each well contained 200 μL of parasite suspension at 1.5% hematocrit and different drug concentrations. The plates were maintained for 40–46 h at 37°C in a candle jar as previously described [27]. Parasite growth was stopped by freezing at −20°C for at least 3 hours.

### *In vitro* drug sensitivity assay

The double-site enzyme-linked lactate dehydrogenase immunodetection (DELI) assay was used to detect *P. falciparum* growth as previously described [28,29]. Briefly, 100 μL of lysing buffer and an appropriate volume of sample were added to MAb 17E4-precoated wells before incubation with shaking at 37°C for 1 h. The plate was washed five times and 100 μL of biotinylated MAb 19G7 was added to each well at 37°C for 30 min. The plate was washed and 100 μL of peroxidase-labeled streptavidin was added at 37°C for 15 min. The plate was washed and 100 μL of a mixture (v/v) of a peroxidase substrate solution (3,3’,5,5’-tetramethylbenzidine and 0.02% H_2_O_2_) (Kirkegaard and Perry, Gaithersburg, MD) was added and incubated in the dark for 15 min. The reaction was stopped by adding 50 μL of 1 M phosphoric acid. The color reaction was quantified in a spectrophotometer at 450 nm, with a reference at 630 nm. Each experiment was performed three times in triplicate.

### Genetic polymorphisms in *pfmdr1*, *pfcrt* and *pfATPase 6*

DNA was extracted from samples by using the blood DNA OMEGA Bio-Tek E.Z.N.A. method (Omega Bio-Tek, USA). Polymorphisms in *pfcrt, pfmdr1* and *Pfatpase6* were detected by polymerase chain reaction (PCR) amplification followed by mutation-specific restriction endonuclease cleavage. Table 1 shows the primer sets and restriction enzymes used, as previously described [30], [31].

**Table 1:** Sequences of primer sets and restriction enzymes used to characterized polymorphisms

**Genes, Codons**	**Primer names**	**Primers**	**T°C**	**Restriction enzyme**	sizes (bp)*
***Pfmdr1, N86Y***	mdr86D1	TTTACCGTTTAAATGTTTACCTGC	**45**	***Afl III***	**126 +165**
	mdr86D2	CCATCTTGATAAAAAACACTTCTT			
***Pfmdr1, D1246Y,***	mdr1246D1	AATGTAAATGAATTTTCAAACC	**45**	***Bgl II***	**113 + 90**
	mdr1246D2	CATCTTCTCTTCCAAATTTGATA			
***ATPase 6, G110A***	ATP6-110F	CGTTGAACTTATTATATCTTTGTC	**60**	***Mbo II***	**103+94+92+40**
	ATP6-110R	TTTCATATCTAATAAAGTTAACACG			
***ATPase 6, A2694T***	ATP6-2694F	GAATTGTTTTCTGTAGAACTGAAC	**55**	***Tas I***	**142 + 39**
	ATP6-2694R	ATCTGATGCTTCTTTAGCTACC			
***Pfcrt, C72S***	CRT72MS	TTTATATTTTAAGTATTATTTATTTAAGTGGA	**55**	***Mbo I***	**55 + 38**
	76-D2	CAAAACTATAGTTACCAATTTTG			
***Pfcrt, M74I***	CRT745MS	TAAGTATTATTTATTTAAGTGTATGTGTCAT	**55**	***Nla III***	**53 + 31**
	76-D2	CAAAACTATAGTTACCAATTTTG			
***Pfcrt, N75M***	CRT745MS	TAAGTATTATTTATTTAAGTGTATGTGTCAT	**50**	***BspHI***	**53 + 31**
	76-D2	CAAAACTATAGTTACCAATTTTG			
***Pfcrt, 76***	Pfcrt-76A	GCGCGCGCATGGCTCACGTTTAGGTGGAG	**55**	***Apo I***	**136 + 56**
	Pfcrt-76B	GGGCCCGGCGGATGTTACAAAACTATAGTTACC			

sizes* indicate the sizes of fragments generated after restriction enzyme digestions.

AA^1^ = mutation on amino acid sequence.

T°C = hybridization temperature during PCR programme.

### Statistical analysis

IC_50_ values with their 95% confidence intervals (CI) were calculated by using an Emax model available at http://www.antimalarial-icestimator.net, as RE = 100 – [(100*C^γ^ )/(C^γ^ + IC_50_^γ^)], where IC_50_ is the drug concentration inhibiting 50% of parasite activity, γ is a sigmoidicity factor which expresses the steepness of the curve, RE is the relative effect of the drug (in percent, Y-axis), and C is the drug concentration (X-axis). The IC_50_ cut-off values for resistance to chloroquine, mefloquine and monoadhesylamodiaquine were 100 nM, 20 nM, 60 nM respectively, and the cut-off for diminished susceptibility to DHA was 10 nM. Associations between drug sensitivity and parasite gene polymorphisms were evaluated with the Chi square test, and routine hematological parameters were analyzed with Fisher’s exact test implemented with Epinfo software. In all statistical tests, significance was assumed at p<0.05.

## Results

### *In vitro* drug sensitivity of *P. falciparum* isolates

**Figure 1:**
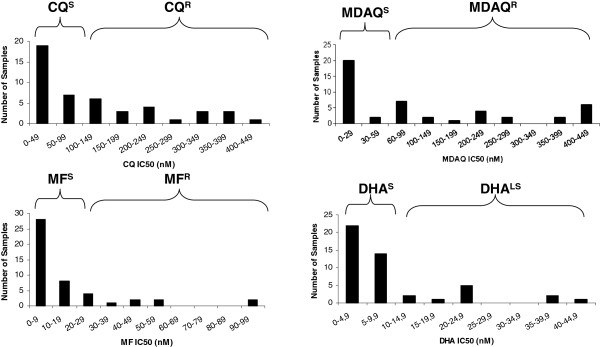
**Sensitivity of clinical samples to CQ, MDAQ, MF and DHA.** Parasites were cultured and drug sensitivities were assessed with the DELI method CQ^R^, CQ^S^, MF^R^, MF^S^, MDAQ^R^, MDAQ^S^, DHA^S^, and DHA^LS^ indicated chloroquine-resistant, chloroquine-sensitive, mefloquine- resistant, mefloquine-sensitive, monodesethylamodiaquine-resistant, monodesethylamodiaquine-sensitive, dihydroartemisinine-sensitive and dihydroartemisinine-low-sensitive.

A total of 53 *P. falciparum* isolates were collected from Franceville hospitals. The IC_50_ values of all four drugs were successfully determined for 46 isolates (Figure 1). The median CQ IC_50_ value was 71.67 (1–438.2) nM, and 43.5% (n=20) of isolates were CQ-resistant. The median MDAQ IC_50_ value was 64.79 (0.09-448) nM, and 52.2% (n= 24) of isolates were MDAQ-resistant. The median mefloquine IC_50_ value was 6.59 (0.08-95.9) nM, and 23.9% (n=11) of isolates were MF-resistant. The median DHA IC_50_ value was 6.45 (0.09-43.17) nM, and 23.9% (n=11) of isolates had reduced DHA susceptibility. Ten isolates were sensitive to all four drugs. A high level of cross-resistance was observed. As shown in Table 2: The activities of DHA and MF showed the strongest correlation (r^2^= 0.73, p< 0.001). Eight of the 11 isolates with decreased DHA sensitivity were also resistant to MF. The activities of CQ and MF also showed a strong correlation (r^2^= 0.45, p=0.04), followed by CQ and DHA (r^2^= 0.39, p=0.006). MDAQ activity did not correlate with the activity of the other three drugs (for DHA: r^2^= 0.37, p=0.08; for CQ: r^2^= 0.34, p=0.5; for MF r^2^= 0.30, p=0.08) (see Table 2).

**Table 2:** Correlation of antimalarial activities

**Drug 1**	**Drug 2**	**Coefficient of correlation (r2)**	**p**
DHA	MF	0.73	< 0.001
DHA	CQ	0.39	0.006
DHA	MDAQ	0.37	0.08
CQ	MF	0.45	0.04
CQ	MDAQ	0.34	0.5
MDAQ	MF	0.30	0.08

### Drug sensitivity and *P. falciparum* gene polymorphisms

Table 3 shows the distribution of polymorphisms at codons 86 and 1246 in *Pfmdr1* according to drug sensitivity. The overall frequencies of the wild-type (N86) *Pfmdr1* and the Y86 mutation were respectively 60.1% (n=28) and 39.1% (n=18). The Y86 mutation was not associated with CQ resistance (30.0% (n=6) in CQ-resistant isolates, and 42.3% (n=11) in CQ-sensitive isolates, p=0.39). The prevalence of the Y86 mutation was respectively 37.5% (n=9) and 45.5% (n=10), 45.5% (n=5) and 34.3% (n=12), and 45.5% (n=5) and 34.3% (n=12) in MDAQ-resistant and MDAQ-sensitive isolates, MF-resistant and MF-sensitive isolates, and DHA-reduced susceptibility and DHA-sensitive isolates. Only 1 mixed genotype (N/Y86) was found, and this isolate was resistant to both MDAQ and CQ. We found no association between the Y86 genotype and drug susceptibility (p>0.26). The overall frequency of the Y1246 mutation was 17.4% in pure genotypes (n=8) and 30.4% (n=14) when combined with mixed D/Y1246. The frequency of Y1246 was not different between CQ-sensitive isolates (23.1%, n=6) and CQ-resistant isolates (10.0%, n=2) (p=0.3). This mutant allele was carried by only 3 MDAQ-resistant isolates (12.5%), and by none of the MDAQ-sensitive isolates. The frequency of Y1246 was 18.2% in MF-resistant isolates but only 5.7% in MF-sensitive isolates (p=0.23). There was no difference in the frequency of this allele between DHA-reduced susceptibility (9.1%) and DHA-sensitive isolates (5.7%) (p=0.53). The frequency of the CVIET haplotype of *Pfcrt* was high (90-100%) (Table 4). Only one isolate sensitive to CQ and MF and resistant to MDAQ carried the CVIEK haplotype. No association was found between this genotype and drug sensitivity (0.23≤p≤0.58). All the isolates were *PfATPase 6* G110. The prevalence of the T2694 mutant of *PfATPase 6* was 26.1% (n=12) overall and did not differ between sensitive and resistant isolates (0.40≤p≤0.60). The frequency of T2694 was respectively 25.0% (n=5) and 19.2% (n=5); 26.9% (n=6) and 15.0% (n=4); 27.3% (n=3) and 20.0% (n=7); and 27.3% (n=3) and 20.0% (n=7) in CQ-resistant and CQ-sensitive isolates; MDAQ-resistant and MDAQ-sensitive isolates; MF-resistant and MF-sensitive isolates; and DHA-reduced susceptibility and DHA-sensitive isolates (Table 5).

**Table 3:** Distribution of PfMDR1 polymorphism according the status drug sensitivity groups

**Drug**	**Profils**	**N**	**N° (%), of strains with polymorphism : (Pfmdr)**							
			**N86Y**				**D1246Y**			
			**N**	**Y**	**MIX**	**p**	**D**	**Y**	**MIX**	**p**
CQ	Resistant (IC50>100 nM)	20	13 (65.0%)	6 (30.0%)	1 (5.0%)	0.39	14 (70.0%)	2 (10.0%)	4 (20.0%)	**0.3**
	Sensitive (IC50<100 nM)	26	15 (57.7%)	11 (42.3%)	0 (0.0%)		18 (69.2%)	6 (23.1%)	2 (7.7%)	
MDAQ	Resistant (IC50 ≥ 60 nM)	24	14 (58.3%)	9 (37.5%)	1 (4.2%)	0.28	16 (66.7%)	3 (12.5%)	5 (20.8%)	**0.09**
	Sensitive (IC50 < 60 nM)	22	12 (54.5%)	10 (45.5%)	0 (0.0%)		20 (90.9%)	0 (0.0%)	2 (9.1%)	
MF	Resistant (IC50>20 nM)	11	6 (54.5%)	5 (45.5%)	0 (0.0%)	0.7	7 (63.6%)	2 (18.2%)	2 (18.2%)	0.23
	Sensitive (IC50<20 nM)	35	22 (62.8%)	12 (34.3%)	1 (2,9%)		28 (80.0%)	2 (5.7%)	5 (14.3%)	
DHA	Reduced susceptibility (IC50 ≥ 10 nM)	11	6 (54.5%)	5 (45.5%)	0 (0.0%)	0.7	9 (81.8%)	1 (9.1%)	1 (9.1%)	0.53
	Sensitive (IC50<10 nM)	35	22 (62.8%)	12 (34.3%)	1 (2.9%)		27 (77.1%)	2 (5.7%)	6 (17.1%)	

**Table 4:** Distribution of PfCRT polymorphism according to the drug sensitivities

**Drug**	**Profils**	**N**	**N° (%), of strains with polymorphism : (Pfcrt)**			
			**HAPLOTYPE 72-76**			
			**CVIEK**	**CVIET**	**MIX**	**p**
CQ	Resistant (IC50 ≥ 100 nM)	20	0 (0.0%)	20 (100.0%)	0 (0.0%)	**0.56**
	Sensitive (IC50 < 100 nM)	26	1 (3.8%)	25 (96.2%)	0 (0.0%)	
MDAQ	Resistant (IC50 ≥ 60 nM)	**24**	**1 (4.2%)**	**23 (95.8%)**	**0 (0.0%)**	**0.29**
	Sensitive (IC50 < 60 nM)	**22**	**0 (0.0%)**	**22 (100%)**	**0 (0.0%)**	
MF	Resistant (IC50 ≥ 20 nM)	11	0 (0.0%)	11 (100.0%)	0 (0.0%)	**0.58**
	Sensitive (IC50 < 20 nM)	35	1 (2.9%)	34 (97.1%)	0 (0.0%)	
DHA	Reduced susceptibility (IC50 ≥ 10 nM)	11	1 (9.1%)	10 (90.9%)	0 (0.0%)	**0.23**
	Sensitive (IC50 < 10 nM)	35	0 (0.0%)	35 (100.0%)	0 (0.0%)	

**Table 5:** Distribution of A2694T PfATPase 6 polymorphism in drug sensitive groups

**Drug**	**Profils**	**N**	**Strains with polymorphism PfATPase 6 A2694T**			
			**A**	**T**	**MIX**	**p**
CQ	Resistant (IC50>100 nM)	20	14 (70.0%)	5 (25.0%)	1 (5.0%)	0.45
	Sensitive (IC50<100 nM)	26	20 (76.9%)	5 (19.2%)	1 (3.8%)	
MDAQ	Resistant (IC50 ≥ 60 nM)	24	17 (69.2%)	6 (26.9%)	1 (3.8%)	0.6
	Sensitive (IC50 < 60 nM)	22	17 (80.0%)	4 (15.0%)	1 (5.0%)	
MF	Resistant (IC50>20 nM)	11	7 (63.6%)	3 (27.3%)	1 (9.1%)	0.4
	Sensitive (IC50<20 nM)	35	27 (77.1%)	7 (20.0%)	1 (2.9%)	
DHA	Reduced susceptibility (IC50 ≥ 10 nM)	11	8 (72.7%)	3 (27.3%)	0 (0.0%)	0.4
	Sensitive (IC50<10 nM)	35	26 (74.3%)	7 (20.0%)	2 (5.71%)	

## Discussion

This study shows that, despite the change in national antimalarial policy, the prevalence of CQ-resistant and MF-resistant isolates in Franceville, Gabon, remains stable compared with the year 2000 [18]. MDAQ resistance has not previously been investigated in Franceville. The level of MDAQ resistance found here is higher than that previously found in Libreville, Gabon, where 100% of isolates were susceptible to MDAQ in the year 2003 [32]. Compared with previous data from southeast of Gabon, where only 5.4% of isolates were MDAQ-resistant [33], we found an high prevalence of *in vitro* MDAQ resistance. The mean MDAQ IC_50_ found here is similar to that reported in Kampala, Uganda, between 2006 and 2008, after implementation of the ACT treatment policy [34]. The high level of MDAQ resistance found here could be explained by fact that amodiaquine is combined with artesunate in one of the recommended ACTs used in Gabon.

We found a high prevalence of isolates with reduced susceptibility to DHA. But, because of the brief action of DHA, this prevalence may have been overestimated. The decrease in DHA susceptibility may have arisen through the use of artemether or artesunate monotherapy to treat uncomplicated malaria, contrary to the recommendations of the Gabonese national program against malaria. Our results are in keeping with data from the capital, Libreville, where the prevalence of artemether resistance was 14%, with a range of 0.8 to 34.8 nM (mean IC_50_ 5.0 nM) [35]. The frequency of isolates with reduced susceptibility to DHA found here is similar to that described in Lambarené, a town situated in central Gabon [36]. We found a strong correlation between decreased susceptibility to DHA and resistance to MF and CQ. These data are consistent with previous data showing that the selection of MF resistance leads to increased resistance to artemisinin in rodent model [37], despite the fact that these drugs do not belong to the same class. Indeed, MF was used before implementation of artemisinin derivates. The correlation between MF and CQ resistance is also consistent with previous reports of cross-resistance [38]. In this case, CQ was introduced first, leading to the resistance to MF. This cross-resistance between these drugs warrants reinforced surveillance of antimalarial drug resistance in Gabon. Surprisingly, MDAQ resistance did not correlate with CQ resistance, despite the facts that MDAQ and CQ belong to the same drug family and that MDAQ and CQ antimalarial activities correlate with each other [34]. However, this lack of correlation might also be explained by a lack of statistical power in our study.

Seven isolates (11.3%) could not be cultured, possibly because of the presence of antimalarial drugs in the patients’ blood that was consistent with manufacture informations.

Previous reports indicate that the use of artemether-lumefantrine is associated with the selection of P*fmdr1*-N*86* wild-type parasites, which are tolerant of low LM concentrations [15,39-41]. The present data confirm those of our previous study showing an increase in *Pfmdr1*-N86 genotype field isolates in Franceville after implementation of the ACT treatment policy [21]. In comparison with the prevalence of N86 in 2004 (15.6%) and 2009 (31.3%), this gradual increase calls for reinforced surveillance of artemether-lumefantrine resistance.

A correlation between the *Pfmdr1* Y86 and Y1246 mutations and both CQ and MDAQ resistances has been reported [34,42], but we did not find this correlation. Surprisingly, the *Pfmdr1* N86Y polymorphism was not associated with decreased sensitivity in this study. This is, however, consistent with data from Lambarené (central Gabon), where Y86 was not found to be associated with CQ resistance in 1998 [43], before implementation of ACT: in this latter study the prevalence of N86 was 20.5% and the Y1246 mutation was not detected. Our data suggest the spread of the Y1246 mutation in Gabon. The 184F mutation was not analysed, as its impact on drug resistance is controversial [14,44], but this mutation has been identified as an independent marker of decreased lumefantrine susceptibility [45] and it will be interesting to investigate it in our study region.

The *Pfmdr1* copy number was not investigated here, as it has not been consistently linked to ACT treatment failure [22,31], and this is one of limitations of our study. Indeed, it has been shown that increased a *Pfmdr1* copy number is associated with reduced *in vitro* susceptibility to mefloquine, lumefantrine and artesunate [22].

While *Pfcrt* CVIET is the most prevalent haplotype in Africa, the SVMNT haplotype has recently migrated to Tanzania and Angola [46,47]. This haplotype, which has relatively little fitness cost, has been associated with emergence of AQ resistance [48,49]. Thus, it is crucial to monitor *Pfcrt* codons 72–76 in all countries in which AQ combination therapy is recommended. Because of human migration between Angola and Gabon, we investigated the *Pfcrt* haplotype 72–76 in our isolates: only haplotypes CVIET and CVIEK were detected. The high prevalence of CVIET (n=45/46) made it difficult to show a significant difference in the distribution of polymorphisms between resistant and sensitive isolates. This haplotype is associated with resistance against 4-aminoquinoline. The persistently high frequency of CVIET, despite the withdrawal of CQ in Gabon, could be due to use of amodiaquine as a partner in some ACTs. Recently, CVIET was also reported to be associated with reduced sensitivity to new 4-aminoquinolines such as piperaquine [50].

Polymorphisms in the putative drug transporter *PfATPase6* were not closely linked to *in vitro* drug sensitivity, in keeping with data from Cameroon, a neighboring country [51]. No mutations were found at codon 110. The E431K, A623E, and S769N polymorphisms were not analysed, because of their rarity in sub-Saharan Africa and their lack of impact on *in vitro* drug resistance [51-53].

## Conclusion

Our results confirm that Franceville, Gabon, is an area with high levels of *P. falciparum in vitro* drug resistance and a particularly high frequency of reduced DHA sensitivity. Polymorphisms associated with drug resistance are highly prevalent. This underlines the need for *in vitro* and molecular surveillance of antimalarial drug resistance.

## Competing interests

The authors declare that they have no competing interests.

## Authors' contributions

RZ conducted this study and participated in the writing of this paper, JBLD conceived this work, conducted study and wrote the article, FL participated in the study as a technician, UB participated in the study as a clinician, EBN participated in the statistical analyses, and in the writing of the paper, and FSTN coordinated the realization of the study and writing of the paper. All authors read and approved the final manuscript.

## Pre-publication history

The pre-publication history for this paper can be accessed here:

http://www.biomedcentral.com/1471-2334/12/307/prepub
